# Determinants of hazardous alcohol use among pregnant women attending antenatal care at public health facilities in Gondar town, Northwest Ethiopia: A nested case-control study

**DOI:** 10.1371/journal.pone.0253162

**Published:** 2021-07-01

**Authors:** Alemu Earsido Addila, Telake Azale, Yigzaw Kebede Gete, Mezgebu Yitayal

**Affiliations:** 1 Department of Public Health, College of Medicine and Health Sciences, Wachemo University, Hossana, Ethiopia; 2 Department of Epidemiology and Biostatistics, Institute of Public Health, College of Medicine and Health Sciences, University of Gondar, Gondar, Ethiopia; 3 Department of Health Education and Behavioral Sciences, Institute of Public Health, College of Medicine and Health Sciences, University of Gondar, Gondar, Ethiopia; 4 Department of Health Systems and Policy, Institute of Public Health, College of Medicine and Health Sciences, University of Gondar, Gondar, Ethiopia; Universita degli Studi di Brescia, ITALY

## Abstract

**Background:**

Alcohol use during pregnancy has a potential negative impact on the health of women and children. Binge or hazardous drinking may do greater alcohol-related damage to the developing fetus than drinking a comparable amount spread over several days or weeks. This study aimed to identify determinants of hazardous alcohol use among pregnant women attending antenatal care at Gondar town public health facilities, Northwest Ethiopia.

**Methods:**

An unmatched facility-based nested case-control study was carried out to identify the determinants of hazardous alcohol use among pregnant women within a prospective cohort study from 29 October 2019 to 7 May 2020. A two-stage random sampling technique was used to select 455 (113 cases and 342 controls) pregnant women. Data collection was performed using the AUDIT-C standardized and pretested questionnaire. Bivariable and multivariable logistic regression analyses were computed to identify the predictors of alcohol consumption using the odds ratio, 95% CI, and p-value < 0.05.

**Results:**

Multivariable logistic regression model revealed that no formal education of the husbands [AOR = 2.79; 95%CI: 1.24, 6.29], being housewife[AOR = 2.43; 95%CI: 1.12, 5.26], poor household wealth index[AOR = 2.65; 95%CI: 1.07, 6.54], unplanned pregnancy [AOR = 4.36;95%CI: 2.44, 7.79], poor social support [AOR = 4.9;95%CI: 2.4, 10.04], depression[AOR = 3.84;95%CI: 2.16, 6.82], and not ever heard the risk of alcohol drinking during pregnancy [AOR = 1.97; 95%CI: 1.08, 3.58] were significantly associated with hazardous alcohol use.

**Conclusions:**

Routine alcohol screening during ANC visits creates an appropriate referral system for clinical management and provides an opportunity for healthcare workers to offer information on the potential risks associated with alcohol use in pregnancy. Antenatal care providers have a special role to play in assuring that women receive adequate advice about alcohol use and care to manage the problems especially for pregnant women with depression, poor social support, unplanned pregnancy, low socioeconomic status, and for housewives during the antenatal visits. The warning marks on alcoholic beverages including an ongoing message about the risks of alcohol use during pregnancy could be public health good strategies to minimize preventable harms attributed to alcohol consumption during pregnancy.

## Background

Alcohol consumption causes death higher than that resulting from other diseases like HIV/AIDS, tuberculosis, and diabetes [1]. Alcohol use is a well-known single largest behavioral risk factor for disease and disability in middle-income countries [2]. Women’s alcohol consumption has risen over time with increasing gender equality and changing gender roles [3]. Alcohol use during pregnancy has potential acute and chronic negative health consequences on the health of women and children. The effect of low to moderate levels of alcohol consumption during pregnancy on the fetus is controversial [4–6], and it is argued that erroneous data on alcohol intake may produce these contradictory results [7]. Moreover, a safe level of alcohol use during pregnancy has not been ascertained and there is no precise dose-response relationship between the amount of alcohol used during the pregnancy and the extent of the problem caused by alcohol in the infant [4, 8]. However, there are strong shreds of evidence that support the association between high prenatal alcohol exposure and adverse pregnancy outcomes such as spontaneous abortion, intrauterine growth retardation, premature birth, stillbirth, and low birth weight which can also result in a range of lifelong irreversible health impact- fetal alcohol spectrum disorders (FASD) [4, 9–11]. Accordingly, evidences suggest that pregnant women and women who want to become pregnant should not drink any amount of alcohol [12]. Furthermore, many medical societies propose not to take any amount of alcohol during pregnancy [13–15].

Despite the guidelines, studies have reported that binge drinking alcohol among pregnant women is a common drinking pattern in many parts of the worldwide including some Africa countries [16, 17]. Binge drinking (≥4 drinks on a single occasion) is the most hazardous pattern of alcohol drinking that can cause high blood alcohol concentration and damages the unborn fetus by passing across the placenta [18]. Binge or hazardous drinking may do greater alcohol-related damage to the developing fetus than drinking a comparable amount spread over several days or weeks because peak blood alcohol concentration is the critical factor [19, 20]. A study has shown that 2.4% of the pregnant women reported drinking six or more units on one occasion at least weekly or monthly and 5.4% of the women reported hazardous drinking with an Alcohol Use Disorders Identification Test–Consumption(AUIDT-C)score of three or more, and 2.2% of them reported risky drinking with Tolerance, Annoyance, Cut Down and Eye-Opener (T-ACE) score of two or more [21]. Only a few studies have been conducted in Sub-Sahara Africa on alcohol exposure during pregnancy, for instance, the prevalence of binge drinking during pregnancy among the general population in South Africa (3.8%) [22], the Democratic Republic of the Congo (24.5%) [23] and Uganda (9%) [24]. According to the studies conducted in Ethiopia, 7.7%of the respondents consumed four to five standard drinks and 2% of the respondents had eight or more drinks on a single occasion [25] and 16.1% had risky alcohol use behavior [26]. However, the determinants of these heavy drinking alcohols have not been addressed well. Even though alcohol consumption during pregnancy leads to major physical, mental, and psychological problems in infants and children, recent trends indicate that hazardous alcohol drinking during pregnancy is becoming one of the pressing most important public health concerns in developing countries.

With booming marketing of industrially-manufactured branded alcoholic beverages over time along with the rising purchasing power of the society in Ethiopia [27], a large proportion of pregnant women consume alcoholic beverages [25, 26, 28, 29]. Furthermore, traditional homemade alcoholic beverages (*Tella*, *Tej*, *Areke*, *Borde*, *and Korofe*) are familiar in the study area and everyone uses them without any restriction [30]. Information on alcohol consumption level and temporal pattern of drinking are very crucial for policymakers to take appropriate measures or interventions [31].

It is clear that different factors may influence individuals to drink alcohol in a different amount of drinking, however; it is essential to know reliable factors for appropriate intervention. Hazardous alcohol use during pregnancy is a major preventable negative risk factor for the developing fetus. To identify such use during the antenatal care system and initiate measures that can avoid undesirable outcomes for both the mother and the child, identifying determinants of hazardous alcohol use is a key preliminary point. Appropriate screening strategies and identifying determinants may facilitate early recognition and intervention for affected individuals and reduce the occurrence of other cases. Meanwhile, timely interventions have long-term advantages for a mother and child. However, there is a paucity of evidence on the determinants of hazardous alcohol consumption during pregnancy in Ethiopia in general. By examining the determinants of hazardous alcohol consumption in the study area, the current study could make a novel contribution to help fill the gaps in the current literature and inform future policy efforts in Ethiopia.

## Materials and methods

### Study design, study setting, and period

We carried out an unmatched facility-based nested case-control study within a prospective cohort study of the effect of alcohol consumption during pregnancy on perinatal adverse health outcomes in Gondar town, Northwest Ethiopia (will be disseminated separately) [32]. It was conducted in selected four public health facilities (one hospital and three health centers), namely the University of Gondar Comprehensive Specialized Hospital, Gondar Polyclinic, Azezo Health Center, and Maraki Health Center in Gondar town from 29 October 2019 to 7May 2020. Gondar town is located about 727 km far from Addis Ababa, the capital city of Ethiopia. In 2018, according to the Gondar town finance and economic development branch office report, the population of Gondar town was approximately 338,646 (165, 937 males and 172, 709 females). Of these females, 7,454 were estimated to be pregnant women. In the town, there are eight health centers and one comprehensive specialized hospital [33]. There is one beer factory in the town. Besides, almost all locally brewed and common other types of alcoholic drinks are available in the town.

### Sample size determination and sampling procedure

We determined the sample size using EPI INFO version 7.2.1.0 STAT CALC software as described by Fleiss with continuity correction to estimate the sample size. From the total cohort of 1,778 pregnant women, we included 113 cases and 342 controls who met all inclusion and exclusion criteria based on the following assumptions: two-sided 95% confidence level, power of 80%, the ratio of sample size 3:1 to detect an odds ratio of at least 0.48 for the determinant of hazardous alcohol use among pregnant women who used to consume alcohol. Evidence shows that the odds ratio of completed secondary educational attachment was 0.48(women who were completed secondary educational attainment were less likely to experience hazardous drinking as compared to primary ones),where the proportion rate of completed secondary in the non-exposed group was 34.04% [34]. We used a two-stage sampling technique to recruit pregnant women and to include them in the cohort. In the first stage, we selected three health centers using a simple random sampling technique and we took one hospital purposively; in the second stage, we chose pregnant mothers who fulfilled the inclusion criteria using a systematic random sampling technique. We proportionally allocated the sample size to each health facility based on previous client-flow information.

### Study variables

#### Response variable

The response variable of the study was hazardous alcohol use during pregnancy which was categorized as “cases” and “controls”.

#### Explanatory variables

The explanatory variables of the study were the age of women, marital status, religion, women’s education, and husbands’ education, family size, occupation, household wealth status, depression, social support, parity, number of children, history of pre-pregnancy alcohol consumption, ever heard the risk of alcohol use, the pattern of the current pregnancy, a perception that alcohol consumption is culturally or socially acceptable, partner alcohol consumption, and antenatal care (ANC) visit.

### Participant selection and recruitment

Women were enrolled if they were in the first week of the third trimester or 28^th^weeks of gestational age. All pregnant women who used to consume alcohol in Gondar town were the source population, and all systematic randomly selected pregnant women who used to consume alcohol in the selected health facilities were the study population. For this nested case-control approach, cases were pregnant women who used to drink hazardous alcohol while controls were pregnant women who used to drink alcohol at a non-hazardous level. Cases were consecutively selected among alcohol drinking pregnant women and the next three corresponding controls were selected by simple random method. Hazardous alcohol use was defined as a pattern or quantity of alcohol consumption during pregnancy with the AUDIT-C score of three or more [21].

### Data collection method and tool

This study was part of a large prospective cohort study, where a similar data collection tool was used in articles published elsewhere [32, 35].The questionnaire was prepared first in English and then translated into Amharic (local language) to suit local applicability and then back to English to ensure its consistency. The tool was developed by reviewing different previous studies of similar objectives [25, 36–41] and it was depicted as (S1 and S2 Tables). Experts’ consultation was sought to ascertain the tool’s validity taking into consideration the local situation of the study participants and clinical relevance. BSc nurses and midwives were data collectors who conducted face-to-face interviews using a structured and pre-tested interviewer administrated questionnaire. Data collectors and supervisors were adequately trained for two days on data collection tools, procedures during data collection, a way how to obtain consent from participants and not to miss any questions in the questionnaire. The Amharic version questionnaire was pre-tested for clarity. It was more pretested through a pilot study of 67 pregnant women in Bahir Dar town which is 180 km far away from the actual study area. The tool was checked for its reliability and validity before actual data collection. To assure data truthfulness, in addition to daily supervision, weekly meetings were conducted with supervisors and data collectors to observe the quality, status, and issues in collecting data.

The alcohol use screening tool, AUDIT-C [40, 42], is the most popular shortened version of the 10-item AUDIT. It comprises of three items to assess alcohol consumption cross-culturally and identify hazardous drinkers [43, 44]. We used the Edinburgh Postnatal Depression Scale (EPDS) which has 10 items scored on a scale of 0–3; the score ranging from 0–30 and we used a cut-off point of 13 and above on the scale to identify women with depressive symptoms [45]. We also usedthe Oslo 3-items social support scale, which is considered one of the best predictors of mental health, covering different fields of social support and perceived ways of getting assistance from neighbors [46, 47]. The sum ranges from 3–14 in which the score of 3–8 shows poor support, 9–11 shows moderate support, and 12–14 shows strong support. The socio-economic status of the households (wealth index) was assessed using 16 variables extracted from Ethiopia Demographic and Health Survey 2016 and Principal Component Analysis was computed to determine it.

### Alcohol consumption measures

The questionnaire was adjusted by considering the local context of alcoholic beverages of alcohol content and drinking containers. Though the amount of alcohol content in a standard drink varies from country to country, we used the WHO’s standard for this study, since Ethiopia has no national alcohol policy defining standard alcohol drinks [1]. Based on this, for a standard drink, 12g of absolute alcohol was assumed to consider as alcohol consumption. A standard drink was determined by converting local drinks to grams of pure alcohol, and then we specified the amount of pure alcohol for each local drink and using local units of measure. Different receptacles(containers) were used to measure local drinks, such as ‘*tassa’*, *malekia’* and ‘*birille’* for drinks *Tella* (the Ethiopian traditional beer made of mostly from barley but also from wheat, maize, sorghum, and mixed with ‘*Gesho’* [Rhamnusprinioides]) [48], *Areki* (a whiskey-like drink distilled from fermented barley or maize and mixed with ’[Rhamnusprinioides]) and *Tej* (a honey wine) respectively. The amount of each drink consumed in ml was then calculated. This value was converted to grams of absolute alcohol by applying a conversion factor and taking into account the percentage of absolute alcohol present in each drink. Accordingly, a standard drink equivalent to 1 bottle beer (330 ml) at 5% x (strength) 0.79 (conversion factor) = 13 grams of ethanol; 1 glass wine (140 ml) at 12% x 0.79 = 13.3 grams of ethanol; 1 shot (‘*malekia’*) *Areki*(40ml) at 40% x 0.79 = 12.6 grams of ethanol, alcoholic content (30–50%); 1 ‘*birille’Tej* (200ml) at 8% x 0.79 = 12.64 gram of ethanol, alcoholic content *(*7%- 11%);and 1 “*tassa*” *Tella/Korofe* (330-500ml) at 4.5% x 0.79 = 11.73 gram of ethanol of alcoholic content (4% - 6%) [49–51].

### Statistical analysis

The data were double entered and edited into EpiData 3.1.version and then exported to STATA version 14 software packages for analysis. To examine the association between various factors with hazardous alcohol drinking, we performed bivariable and multivariable logistic regression analysis. The explanatory variable whose bivariable test had a *p*-value ≤0.25 was considered a candidate for the multivariable model along with all variables of clinically known importance. The strength of association was presented using crude odds ratio (COR) and adjusted odds ratio (AOR) with corresponding 95% confidence interval (CI) and the *p*-value < 0.05 was set to declare the statistical significance threshold in the multivariable analysis. The Hosmer and Lemeshow goodness-of-fit test was used to see whether our model adequately described the results, and it was not statistically significant in this case. The occurrence of multicollinearity among explanatory variables was checked using the Variance Inflation Factor (VIF) at a cut-off point of 10 [52].The interaction effect of the variables was checked by creating the product term and then a new variable became either statistically significant or not at p-value <0.05. However, there was no interaction between the independent variables.

### Ethical considerations

We obtained ethical clearance from the Institutional Ethical Review Board of the University of Gondar (R. No.-O/V/P/RCS/05/747/2019) and we got permission from Amhara Public Health Institute and Gondar town Health Department before the commencement of the study. Before enrolment of the participants, we informed all respondents about the importance of the study, its objective, effects, and the significance of participation. We also obtained verbal informed consent before conducting data collection and we kept all information anonymously in order to maintain confidentiality. After taking the necessary information, participants were counseled about the risks of alcohol drinking during pregnancy and advised not to drink any alcohol during pregnancy or while trying to get pregnant. Besides, women who were engaged in hazardous drinking were referred to healthcare providers and proper linkage was established to get possible treatment options in their respective health facilities.

## Results

### Socio-demographic and economic characteristics of the respondents

A total of 342 women in the non-hazardous alcohol use group and 113 in the hazardous alcohol use group were included in this study. The mean (± standard deviation) age of the respondents was 26.87(±4.5) years for cases and26.47 (±4.3) years for controls. Around two-thirds of the study participants (64.60%of cases and 63.45% of controls) were in the age group of between 25 and 34 years for cases and controls. Most of the study participants of the cases (100%) were orthodox compared with the controls (97.66%). Concerning the main occupation, 64(56.64%) of cases and 148(43.27%) of controls were housewives (Table 1).

**Table 1:** Socio-demographic and economic characteristics of pregnant women attending ANC at Gondar town public health facilities, Northwest Ethiopia, 2020 (n = 455).

Characteristics	Cases = 113	Controls = 342	P-value
	n(%)	n(%)	
**Age group (years)**			0.587
15–24	30(26.55)	103(30.12)	
25–34	73(64.60)	217(63.45)	
≥35	10(8.84)	22(6.43)	
**Marital status**			0.400
Married	111(98.23)	331(96.78)	
Single/divorced/separated/widowed	2(1.77)	11(3.22)	
**Religion**			
Orthodox	113(100)	334(97.66)	
Muslim	0(0.00)	2(0.58)	
Protestant	0(0.00)	6(1.75)	
**Ethnicity**			0.264
Amhara	111(98.23)	329(96.20)	
Others	2(1.77)	13(3.80)	
**Education level of respondents**			0.373
No formal education	32(28.32)	72(21.05)	
Primary education (1–8)	17(15.04)	56(16.37)	
Secondary education(9–12)	36(31.86)	108(31.58)	
Tertiary education(above 12)	28(24.78)	106(30.99)	
**Education level of husbands**			0.005
No formal education	28(24.78)	52(15.20)	
Primary education(1–8)	18(15.93)	35(10.23)	
Secondary education(9–12)	36(31.86)	105(30.70)	
Tertiary education(above 12)	31(27.43)	150(43.86)	
**Main occupation**			0.004
Housewife	64(56.64)	148(43.27)	
Employed in any organization	30(26.55)	91(26.61)	
Merchant	12(10.62)	87(25.44)	
Others	7(6.19)	16(4.68)	
**Household wealth status**			0.027
Poorest	9(7.96)	62(18.13)	
Poor	32(28.32)	67(19.59)	
Middle	19(16.81)	73(21.35)	
Rich	25(22.12)	69(20.17)	
Richest	28(24.78)	71(20.76)	

### Reproductive and medical history related characteristics of study participants at Gondar town public health facilities

The pattern of the current pregnancy was planned in 70(61.95%) of cases and 298(87.13%) of controls. Concerning social support, only 10.62% of the cases and 37.43% of the controls had strong social support. An almost equal proportion of the cases (38.05%) and controls (38.01%) had the perception that alcohol use is culturally or socially acceptable. Very few study participants, 13.27% of the cases and 23.10%of the controls, reported that they were informed about the risk of alcohol use during pregnancy at ANC visit (Table 2).

**Table 2:** Reproductive and medical history related characteristics of study participants attending ANC at Gondar town public health facilities, Northwest Ethiopia, (n = 455).

Characteristics	Cases = 113	Controls = 342	P-value
	n (%)	n (%)	
**Parity**			0.746
Nulliparous	43(38.05)	136(39.77)	
Multiparous	70(61.95)	206(60.23)	
**The pattern of the current pregnancy plan**			<0.001
Planned	70(61.95)	298(87.13)	
Unplanned	43(38.05)	44(12.87)	
**Number of children**			0.562
No child yet	42(37.17)	139(40.64)	
1–2 children	63(55.75)	172(50.29)	
≥3	8(7.08)	31(9.06)	
**History of abortion**			0.788
Yes	16(14.16)	45(39.82)	
No	97(85.84)	297(61.18)	
**Social support**			<0.001
Poor	80(70.80)	156(45.61)	
Moderate	21(18.58)	58(16.96)	
Strong	12(10.62)	128(37.43)	
**Depression**			<0.001
Yes	43(38.05)	58(16.96)	
No	70(61.95)	284(83.04)	
**History of pre-pregnancy alcohol use**			0.247
Yes	80(70.80)	222(64.91)	
No	33(29.20)	120(33.09)	
**Partner encouragement to alcohol use**			0.592
Yes	6(5.30)	14(4.09)	
No	107(94.70)	328(95.91)	
**The perception that alcohol use is culturally or socially acceptable**			0.993
Yes	43(38.05)	130(38.01)	
No	70(61.95)	212(61.99)	
**Ever heard of the risk of alcohol drinking during pregnancy**			<0.001
Yes	26(23.00)	146(42.69)	
No	87(77.00)	196(57.31)	
**Informed the risk of alcohol use at ANC visit**			0.020
Yes	15(13.27)	79(23.10)	
No	98(86.73)	263(76.90)	

### Determinants of hazardous alcohol use among pregnant attending antenatal care at Gondar town public health facilities

The present study revealed that husbands’ educational level, main occupation, household wealth index, the pattern of the current pregnancy plan, social support, depression, and ever heard about the risk of alcohol drinking during pregnancy were significantly associated with hazardous alcohol use at p-value < 0.05. In the analysis, after adjusting for possible confounding variables, the study participants who had husbands with no formal educational level were almost 2.80 times [AOR = 2.79; 95%CI: 1.24, 6.29] more likely to use hazardous alcohol than those who had husbands with a tertiary education level. The odds of hazardous alcohol use among study participants who were housewives were 2.4 times [AOR = 2.43; 95%CI: 1.12, 5.26] higher than those who were merchants. Respondents who were poor in economical status were 2.65 times [AOR = 2.65; 95%CI: 1.07, 6.54] more likely to use hazardous alcohol than the poorest mothers. Likewise, after controlling the effect of other variables, mothers who did not plan their current pregnancy were 4.36 times [AOR = 4.36; 95%CI: 2.44, 7.79] more likely to use hazardous alcohol compared to those who did plan the current pregnancy. The likelihood of hazardous alcohol use during pregnancy among those who had poor social support and depression was 4.91 times [AOR = 4.9; 95%CI: 2.4, 10.04] and 3.84 times [AOR = 3.84; 95%CI: 2.16, 6.82] higher than those who had strong social support and no depression, respectively. Finally, respondents who had not ever heard about the risk of alcohol drinking during pregnancy were nearly two-folds [AOR = 1.97; 95%CI: 1.08, 3.58] higher compared to respondents who heard about the risk of alcohol drinking during pregnancy (Table 3).

**Table 3:** Determinants of hazardous alcohol use among pregnant attending antenatal care at Gondar town public health facilities, Northwest Ethiopia, 2020.

Characteristics	Cases	Controls	COR(95%CI)	AOR(95%CI)
	n(%)	n(%)		
**Education level of husbands**				
No formal education	28(24.78)	52(15.20)	2.6(1.43,4.75)	2.79(1.24,6.29)*
Primary education(1–8)	18(15.93)	35(10.23)	2.49(1.25,4.95)	2.19(0.93,5.13)
Secondary education(9–12)	36(31.86)	105(30.70)	1.66(0.97,2.85)	1.40(0.73,2.70)
Tertiary education (above 12)	31(27.43)	150(43.86)	1	1
**Main occupation**				
Merchant	12(10.62)	87(25.44)	1	1
Housewife	64(56.64)	148(43.27)	3.14(1.60,6.13)**	2.43(1.12,5.26)*
Employed in any organization	30(26.55)	91(26.61)	2.39(1.15,4.97)*	2.23(0.96,5.20)
Others	7(6.19)	16(4.68)	3.17(1.08,9.28)*	2.88(0.81,10.22)
**Household wealth status**				
Poorest	9(7.96)	62(18.13)	1	1
Poor	32(28.32)	67(19.59)	3.29(1.45,7.44)**	2.65(1.07,6.54)*
Middle	19(16.81)	73(21.35)	1.79(0.76,4.25)	1.31(0.49,3.51)
Rich	25(22.12)	69(20.17)	2.50(1.08,5.76)*	2.24(0.87,5.80)
Richest	28(24.78)	71(20.76)	2.72(1.19,6.20)*	1.10(0.42,2.88)
**The pattern of the current pregnancy plan**				
Planned	70(61.95)	298(87.13)	1	1
Unplanned	43(38.05)	44(12.87)	4.16(2.54,6.82)***	4.36(2.44,7.79)***
**Social support**				
Poor	80(70.80)	156(45.61)	5.47(2.86,10.48)***	4.91(2.4,10.04)***
Moderate	21(18.58)	58(16.96)	3.86(1.78,8.38)**	2.20(0.92,5.29)
Strong	12(10.62)	128(37.43)	1	1
**Depression**				
Yes	43(38.05)	58(16.96)	3.01(1.87,4.83)***	3.84(2.16,6.82)***
No	70(61.95)	284(83.04)	1	1
**Ever heard of the risk of alcohol drinking during pregnancy**				
Yes	26(23.00)	146(42.69)	1	1
No	87(77.00)	196(57.31)	2.49(1.53,4.06)***	1.97(1.08,3.58)*
**Informed the risk of alcohol use at ANC visit**				
Yes	15(13.27)	79(23.10)	1	1
No	98(86.73)	263(76.90)	1.96(1.08,3.57)*	1.6(0.81,3.24)

Note:

*** = P-value <0.001

** = P-value <0.01

* = P-value <0.05.

The Hosmer-Lemeshow statistics were not statistically significant i.e. prob>chi^2^ = 0.2151 and this value indicates that the model fits the data reasonably well. In this study, the maximum value of VIF was 3.86, which confirmed the absence of multicollinearity between the explanatory variables.

## Discussion

Hazardous alcohol consumption during pregnancy is a major public health concern that may cause multiple potential risks on the developing fetus and mother. The study revealed that the education level of the husbands, the main occupation of the respondents, wealth status of the household, the pattern of the current pregnancy plan, social support, depression, and ever heard information about the risk of alcohol drinking during pregnancy were the independent determinants of hazardous alcohol consumption during pregnancy.

The results of this study revealed that the likelihood of hazardous alcohol consumption during pregnancy was higher among respondents who had husbands with no formal educational attainment than those with tertiary education levels. The possible explanation for this correlation might be husbands’ educational attainment plays a great role in decision-making power and had an even greater influence than did wives’ education level on maternal health service decisions in developing countries [53–56]. Accordingly, husbands with high education attainments might have adequate knowledge on common health issues including adverse effects of hazardous alcohol use during pregnancy.

Another finding was that the main occupation appears to be an important determinant of hazardous alcohol use. Being a housewife has constituted a greater influence to drink hazardous alcohol than a merchant. The association might be due to the fact that when mothers stay at home regularly for a longer period, they may not have information concerning maternal and child healthcare and self-uncertainty about their ability to be effective women. Since housewives are heavily engaged in taking care of their children, preparing food, and other domestic duties within a restricted environment, they may easily be vulnerable to certain mental health problems like antenatal depression [57, 58] which was a co-occurrence with high alcohol use as evidenced in the result of different studies [26, 59]. Similarly, the poorest pregnant women were more likely to engage in hazardous drinking than those with poor wealth status. Even though there is no great difference between the poorest and poor, this could be attributed to several factors including lack of accessibility to good healthcare and the severity of stressful events. Research findings have shown that alcohol abuse and binge drinking are correlatively much more common in disadvantaged localities than those with higher-income neighborhoods. This might be due to experiencing extreme poverty may impact through leading an unhealthy lifestyle and a direct consequence of poor life circumstances and psychosocial stresses.

We also found that hazardous alcohol use was significantly associated with the pattern of the current pregnancy plan. This finding is consistent with previous studies conducted in various countries [60–62]. For instance, one study indicated that women with an unplanned pregnancy are more often involved in binge drinking during pregnancy than women with a planned pregnancy. The reason for this association could be women with an unplanned pregnancy may suffer from social and psychological crises that can have the potential to drive them to use high alcohol and other substances to get relief from their stressful experiences [63]. The relationship between a higher level of planned pregnancy and lower rates of hazardous drinking is commonly predictable and this adheres with alcohol drinking guidelines for women trying to get pregnant and pregnant.

The odds of hazardous alcohol use during pregnancy were nearly five-folds higher among mothers who had poor social support compared to those who had strong social support. This finding was concordant with the study conducted on risky alcohol use behavior among pregnant women in other settings of Ethiopia [26]; even though, there was variation in the target population. When social support among pregnant women is high, they may share information concerning their pregnancy and other maternal health services which positively play an important role in shaping women’s health-seeking behavior. On the contrary, pregnant women with poor social support may be prone to loneliness and different mental disorders [64] that are commonly interrelated to high alcohol consumption.

Regarding depression, the odds of hazardous alcohol use among pregnant women who had depression were 3.84 times higher compared to those of their counterparts. In spite of the difference in the source population, this finding was in line with other previous studies including systematic review and meta-analysis [26, 28, 59]. High rates of co-morbidity between alcohol use disorders and depression have been documented in epidemiological studies [65] and pregnant women with depression are also more likely to engage in high drinking [66]. Moreover, those with alcohol use disorder may often drink too much alcohol. This linkage could be due to pregnant women may use hazardous alcohol as a form of self-medication to get relief against depression.

Finally, this finding also indicated that information about the risk of alcohol consumption during pregnancy had a significant association with hazardous alcohol use. The likelihood of hazardous alcohol use among pregnant women who had not ever heard about the risk of alcohol drinking during pregnancy was almost two times higher compared to their counterparts. The likely reason for this association could be pregnant women who are exposed to different messages about the adverse effect of alcohol consumption during pregnancy may create some awareness and drink small quantities. Previous research found that pregnant women were less likely to consume alcohol under abstinence guidelines when they had adequate information about the risk of alcohol use [67] and the drinking behavior could be attributable to information pregnant mothers received. Even though recommendations of abstinence from alcohol use during pregnancy, only 20.66% of pregnant women had got information about the potential risk of alcohol use during ANC visits by healthcare providers. Pregnant women may have a reluctance to disclose special risk factors.

## Strengths and limitations of the study

Besides proper counseling of all study participants on potential risks of alcohol use during pregnancy, women with hazardous alcohol drinking were linked to healthcare professionals to provide appropriate advice and follow up. Regardless of its strengths, when interpreting and using the results of this study, potential limitations should be taken into consideration. It was an institution-based study that could limit its external validity. This study could be prone to social desirability and recall bias. Exactly categorization of cases and controls might be difficult from the cohort because it was dependent entirely on self-reports. Moreover, ascertainment of the true magnitude and amount of alcohol consumption is difficult as under-reporting is highly expected and women’s understanding of what constitutes a clear standard drink may be complicated. Because of the literature gap in case-control studies, we used cross-sectional studies for comparison purposes; therefore, the result of this study should be interpreted with caution. Finally, even if we adjusted for multiple confounding variables, as with all observational studies, we could not exclude the possibility of some residual confounding factors.

## Recommendations and conclusions

Even though current evidence shows that any amount of alcohol exposure during pregnancy has a potentially adverse effect on the developing fetus [68], the general public considers low to moderate alcohol exposure in pregnancy as having little or no risk. This nested case-control study under the umbrella of the cohort project indicates a great public health implication for policymakers and people who have an enormous interest to work on the prevention of the consequences of alcohol consumption during pregnancy. It has been found that most pregnant women with hazardous alcohol consumption were not detected in antenatal clinics with specific screening. Routine alcohol screening during ANC visits creates an appropriate referral system for clinical management and provides an opportunity for healthcare workers to offer information on the potential risks associated with alcohol use in pregnancy. The warning marks on alcoholic beverages including ongoing education about the risks of alcohol use during pregnancy could be public health good strategy to minimize preventable harms attributed to alcohol consumption during pregnancy. Antenatal care providers have a special role to play in assuring that women receive adequate advice about alcohol use and care to manage including other health risk factors especially for pregnant women with depression, poor social support, unplanned pregnancy, low socioeconomic status, and for housewives during the antenatal visits.

## Supporting information

S1 Table English version questionnaire. (DOCX)

S2 Table Amharic version questionnaire. (DOCX)

S1 Dataset (DTA)
