# Supplementary material for: Determinants of hazardous alcohol use among pregnant women attending antenatal care at public health facilities in Gondar town, Northwest Ethiopia: A nested case-control study
Source: PLoS One. 2021 Jul 1;16(7):e0253162. doi: 10.1371/journal.pone.0253162 (PMC8248645; doi:10.1371/journal.pone.0253162)
Supplement: S1 Table — (DOCX) [file pone.0253162.s001.docx]

**Questionnaire on determinants of hazardous alcohol use among pregnant women**

**Part I- General information:** Please fill-out all the relevant information in the table below.

**Questionnaire ID: ______________**

| **S. No** | **Questions** | **Response** |
| --- | --- | --- |
| 101 | Individual ID |  |
| 102 | Name of data collector |  |
| 103 | Date of interview |  |
| 104 | Name of supervisor |  |
| 105 | Name of Health facility |  |
| 106 | House number |  |
| 107 | Card number of the respondent |  |
| 108 | Cell phone of the respondent |  |

Part II: Socio-demographic and economic characteristics of the respondents.

| No | Questions for respondent | Response of the respondent | Remark |
| --- | --- | --- | --- |
| 2.01 | What is your age? | _________yrs |  |
| 2.02 | What is your marital status? | 1. Married (live with husband/cohabiting) 2. Single (live alone) 3. Widowed 4. Divorced |  |
| 2.03 | How many family members do you have? | -------------------- |  |
| 2.04 | What is your religion? | 1.Orthodox 2.Protestant  3. Muslim 4. Others, specify |  |
| 2.05 | What is your ethnicity? | 1. Amhara 2.Tigre 3.Oromo  4.Others specify |  |
| 2.06 | What is your Educational status? | 1. No formal education  2. Primary education (1-8)  3. Secondary education(9-12)  4. Tertiary education(above 12) |  |
| 2.07 | What is your husband’s educational status? | 1. No formal education  2. Primary education (1-8)  3. Secondary education(9-12)  4. Tertiary education(above 12) |  |
| 2.08 | What is your main occupation? | 1.Student  2.Private Business (merchant)  3.Employed in any organization  4.Farmer  5.House wife  6.other,specify___________ |  |

| **Part III:- Edinburgh Postnatal Depression Scale (EPDS) to screen depression among pregnant women in Gondar town** | | |
| --- | --- | --- |
| 3.01 | In the past week I have been able to laugh and see the funny side of things: | 1. As much as I always could 2. Not quite so much now 3. Definitely not so much now 4. Not at all |
| 3.02   | In the past week I have looked forward with enjoyment to things: | 1. As much as I ever did 2. Rather less than I used to 3. Definitely less than I used to 4. Hardly at all |
| 3.03 | In the past week I have blamed myself unnecessarily when things went wrong: | 3- Yes, most of the time  2-Yes, some of the time  1-Not very often  0-No, never |
| 304 | In the past week I have been anxious or worried for no good reason: | 1. No, not at all 2. Hardly ever 3. Yes, sometimes 4. Yes, very often |
| 3.05 | In the last week I have felt scared or panicky for no very good reason: | 3-Yes, quite a lot  2-Yes, sometimes  1-No, not much  0-No, not at all |
| 3.06 | In the past week things have been getting on top of me: | 3-Yes, most of the time I haven't been able to cope at all  2-Yes, sometimes I haven't been coping as well as usual  1-No, most of the time I have coped quite well  0-No, I have been coping as well as ever |
| 3.07 | In the past week I have been so unhappy that I have difficulty sleeping: | 3- Yes, most of the time  2-Yes, sometimes  1-Not very often  0-No, not at all |
| 3.08 | In the past week I have felt sad or miserable: | 3-Yes, most of the time  2-Yes, sometimes  1-Not very often  0-No, not at all |
| 3.09 | In the past week I have been so unhappy that I have been crying: | 3-Yes, most of the time  2-Yes, quite often  1-Only occasionally  0-No, never |
| 3.10 | In the past week the thought of harming myself has occurred to me: | 3-Yes, quite often  2-Sometimes  1-Hardly ever  0-Never |

| **Part IV:- The Oslo 3-items social support scale to assess social support of the respondents** | | | |
| --- | --- | --- | --- |
| 4.01 | **How many people are so close to you that you can count on them if you have serious problems?** | **Score** |  |
|  | No | 1 |  |
|  | 1 or 2 | 2 |  |
|  | 3-5 | 3 |  |
|  | 6 and above | 4 |  |
| 4.02 | **How much concern do people show in what you are doing?** |  |  |
|  | A lot of concern and interest | 5 |  |
|  | Some concern and interest | 4 |  |
|  | Uncertain | 3 |  |
|  | Little concern and interest | 2 |  |
|  | No concern and interest | 1 |  |
| 4.03 | **How easy can you get practical help from neighbors if you should need it?** |  |  |
|  | Very easy | 5 |  |
|  | Easy | 4 |  |
|  | Possible | 3 |  |
|  | Difficult | 2 |  |
|  | Very Difficult | 1 |  |

**Part V: Reproductive and some medical history of pregnant women/ respondents**

| **No** | | **Questions** | | | | **Response of the respondent** | | | | | | **Remark** | | |
| --- | --- | --- | --- | --- | --- | --- | --- | --- | --- | --- | --- | --- | --- | --- |
| 5.01 | | What is the number of livebirth you delivered? | | | | ----------- | | | | | |  | | |
| 5.02 | | What is the gestational age of your fetus? | | | | 1.----------  2. I am no sure | | | | | |  | | |
| 5.03 | | When did your last menstrual period come? | | | | ----------- | | | | | | See the card for ultrasound result | | |
| 5.04 | | When is your expected delivery date (EDD)? | | | | ---------- | | | | | |  | | |
| 5.05 | | How many children do you have? | | | | 1. No child yet 2. 1-2 3. 3 – 4 4. 5 and above | | | | | |  | | |
| 5.06 | | Is your current pregnancy planned | | | | 1.Yes 2.No | | | | | |  | | |
| 5.07 | | Do you have previous history of preterm birth? | | | | 1. Yes 2.No | | | | | |  | | |
| 5.08 | | Do you have previous history of abortion? | | | | 1. Yes 2.No | | | | | |  | | |
| 5.09 | | Do you have known diabetics mellitus? | | | | 1. Yes 2.No | | | | | |  | | |
| 5.10 | | Do you have known Hypertension before pregnancy or before 20 wks of gestation? | | | | 1. Yes 2.No | | | | | |  | | |
| 5.11 | | Do you have intention to give birth in the future? | | | | 1. Yes 2.No | | | | | |  | | |
| **Part VI:-History of maternal alcohol consumption/The Alcohol Use Disorders Identification Test (AUDIT-C)** | | | | | | | | | | | | | | |
| 6.01 | Do you currently drink coffee? | | - 1. Yes  1. No | | | | | | |  | | | | |
| 6.02 | How many cups of coffee do you drink per day? | | 1. 1- 3 2. 4-7 3. 8 or above | | | | | | | If yes for Q6.01 | | | | |
| 6.03 | Do you currently smoke cigarette? | | - 1. Yes  1. No | | | | | | |  | | | | |
| 6.04 | Do you currently chew khat? | | 1. Yes 2. No | | | | | | |  | | | | |
| 6.05 | When did you drink a unit of alcohol for last time?  **Note: show the symbol of alcohol container card** | | 1. Before pregnancy 2. During pregnancy 3. Never drink   (more than one option) | | | | | | | **A unit of alcohol means**  1. 1 bottle beer  2. 1 glass wine (140ml)  3. 1*malekia* (whisky, *Areki*, gin….(40ml)  4. 1 can*(Birille)* *Tej* (200ml)  5. 1 can (*Tassa*) *Tella*, *Korfe* (330-500 ml) | | | | |
| 6.06 | How often do you have a drink containing alcohol during pregnancy? | | (0) Never  (1) Monthly or less  (2) 2 to 4 times a month  (3) 2 to 3 times a week  (4) 4 or more times a week | | | | | | |  | | | | |
| 6.07 | How many drinks/units containing alcohol do you have on a typical day when you are drinking during pregnancy? | | (0) 1 or 2  (1) 3 or 4  (2) 5 or 6  (3) 7, 8, or 9 (4) 10 or more | | | | | | |  | | | | |
| 6.08 | How often do you have six or more drinks on one occasion? | | (0) Never (1) Less than monthly  (2) Monthly (3) Weekly  (4) Daily or almost daily | | | | | | |  | | | | |
| 6.09 | When and how many times you had consumed four alcoholic drinks on a single occasion since the onset of pregnancy? | | (0) Didn’t drink  (1) First trimester -----------  (2) Second trimester ----------  (3)Third trimester ----------- | | | | | | |  | | | | |
| 6.10 | Tell me the amount and type of drinks during pregnancy | | Tella | Areki | Tej | | Korfe | Beer/ Jambo draft | Wine | | Sprits  (vodka,whisky..) | | | Write the amount of container |
|  | 1. At first trimester | |  |  |  | |  |  |  | |  | | |  |
|  | 1. At second trimester | |  |  |  | |  |  |  | |  | | |  |
|  | 1. At third trimester | |  |  |  | |  |  |  | |  | | |  |
| **Part VII:- Individual &socio-cultural factors of alcohol consumption during pregnancy** | | | | | | | | | | | | | | |
| 7.01 | | Does your partner drink alcohol? | | | | 1. Yes 2.No | | | | | | |  | |
| 7.02 | | Does your partner encourage you to drink alcohol? | | | | 1. Yes 2.No | | | | | | |  | |
| 7.03 | | Does alcohol use encouraged by your peers? | | | | 1. Yes 2.No | | | | | | |  | |
| 7.04 | | Has alcohol drinking during pregnancy been accepted in your culture? | | | | 1. Yes 2.No | | | | | | |  | |
| 7.05 | | Who has played a great role to drink alcohol for you? | | | | 1. Myself 2. My partner 3. My peers | | | | | | |  | |

| **Part VIII: Sources of information about risk of alcohol consumption during pregnancy** | | | |
| --- | --- | --- | --- |
| 8.01 | Have you heard about risks of alcohol consumption during pregnancy? | 1. Yes B. No | If the answer is no go to Q 9.01 |
| 8.02 | What is/are the source of information about risks of alcohol consumption during pregnancy? | 1. TV 2. Radio 3. Health personnel 4. Friend/family/ peers | More than one answer |
| 8.03 | Have you been informed about risks of alcohol drinking during ANC visit? | 1. Yes B. No |  |

**Part XI: Wealth Index for Urban**

| **S.No** | **Questions** | **Answers** | | |
| --- | --- | --- | --- | --- |
| 9.01 | Who is the owner of the house? | 1. Me 2. Rental | | 3. Family 4. Others...... |
| 9.02 | Main material of the roof? | 1. Corrugated sheet 2. Grass | | 1. Plastic sheets 2. Corrugated iron 3. Others---- |
| 9.03 | Main material of the dwelling floor? | 1. Soil/Sand 2. Wood 3. Ceramic tiles | | 1. Cement 2. Stone 3. Others........ |
| 9.04 | Main material of the exterior walls? | 1. Soil /Sand 2. Bricks 3. Cement blocks | | 1. Stone 2. Wood 3. Others......... |
| 9.05 | How many rooms are available in this house? | -------------- | | |
| 9.06 | How many rooms in this house are used for sleeping? | -------------- | | |
| 9.07 | What is the main source of drinking water for members of your household? (More than one answer is possible) | 1. Piped 2. Open well 3. Rain water | 1. Spring 2. River 3. Others.......... | |
| 9.08 | What kind of toilet facility does most members of your household use? | 1. Ventilated improved pit latrine 2. Traditional latrine | 1. No toilet 2. Others…......... | |
| 9.09 | Do you have a separate kitchen? | 1. Yes | 1. No | |
| 9.10 | Does the household have electric power? | 1. Yes | 1. No | |
| 9.11 | What type of fuel does your household mainly use for cooking? (More than one answer is possible ) | 1. Electricity 2. Wood 3. Charkol | 1. Biogas 2. Natural gas 3. Others........... | |
| 9.12 | Does your household have the following materials? (More than one answer is possible ) | 1. Fixed phone 2. Refrigerator 3. Radio | 1. Television 2. Electric mitad 3. Modern bed 7. Others-- | |
| 9.13 | Does any member of the household have the following resources? (More than one answer is possible ) | 1. Bicycle--- 2. Bajaj---- 3. Motor cycle ---- | 1. Car--- 2. Gari ---- 3. Others-- | |
| 9.14 | Does any member of the household have a mobile phone? | 1. Smart---- | 1. Not Smart ---- 3. No | |
| 9.15 | What is the main source of income for the household? | 1. Agriculture 2. Monthly Salary 3. Trade | 4.Family support  5.Daily laborer  6. Others ………… | |
| 9.16 | Does any member of this household have a bank or microfinance saving account? | 1. Yes፤ Number ------- 2. No | | |

##

**Thank you for your response!**
