# Supplementary material for: Determinants of hazardous alcohol use among pregnant women attending antenatal care at public health facilities in Gondar town, Northwest Ethiopia: A nested case-control study
Source: PLoS One. 2021 Jul 1;16(7):e0253162. doi: 10.1371/journal.pone.0253162 (PMC8248645; doi:10.1371/journal.pone.0253162)
Supplement: S2 Table — (DOCX) [file pone.0253162.s002.docx]

**ክፍል አንድ**፡ **በእርግዝና ጊዜ ስለ አልኮል መጠጣት አጠቃላይ መረጀ**

**የቃለ መጠይቁ መለያ ቁጥር--------------**

| **ተ.ቁ** | **ጥያቄዎች** | **ምላሽ** |
| --- | --- | --- |
| 101 | የግለሰቡ መለያ |  |
| 102 | ቃለ መጠይቁን ያደረገዉ ሰዉ ስም |  |
| 103 | ቃለ መጠይቁ የተደረገበት ቀን |  |
| 104 | ቃለ መጠይቁን የገመገመዉ ሰዉ ስም |  |
| 105 | የጤና ድርጅት ስም |  |
| 106 | የቤት ቁጥር |  |
| 107 | ካርድ ቁጥር |  |
| 108 | የተጠያቂዉ ስልክ ቁጥር |  |

**ክፍል ሁለት፡- የማህበራዊ፣ኢኮኖሚያዊ እና ስና ልቦናዊ ሁኔታዎች መጠይቅ**

| **ተ.ቁ** | **ጥያቄዎች** | **የተሰጡ ምላሾች /መልሶች** | **ምርመራ** |
| --- | --- | --- | --- |
| 201 | እድሜዎት ስንት ነዉ? | ---------------- |  |
| 202 | የጋብቻ ሁኔታ እንዴት ነዉ? | 1. ያገቡ/ ከባል ጋር አብረዉ የምኖሩ 2. ብቻዉን የምኖሩ/ያለገበች 3. ባለዋ የሞተባት 4. የፈተች |  |
| 203 | የቤተሰብዎ ቁጥር ስንት ነዉ? | ---------------- |  |
| 204 | ሀይማኖትዎ ምንድን ነዉ ? | 1. ኦርቶዶክስ 3. ሙስሊም 2. ፕሮቴስታንት 4. ሌላ ካለ ይገለፅ ------- |  |
| 205 | ብሔርዎት ምንድን ነዉ ? | 1. አማራ 2. ትግሬ 3. ኦሮሞ 4. ሌለ ካለ ይገለፅ ----- |  |
| 206 | የትምህርት ደረጃዎት ምን ያህል ነዉ? | 1. መደበኛ ት/ት ያልተማረች 2. ከ1-8ኛ የተማረች 3. ከ9- 12ኛ የተማረች 4. ከ12ኛ በላይ የተማረች |  |
| 207 | የባልዎት የትምህርት ደረጃ ምን ያህል ነዉ? | 1. መደበኛ ት/ት ያልተማረ 2. ከ1-8ኛ የተማረ 3. ከ9- 12ኛ የተማረ 4. ከ12ኛ በላይ የተማረ |  |
| 208 | ዋና ስራዎት ምንድን ነዉ? | 1. ተማሪ 2. ነጋዴ 3. በግል ወይም በመንግስት መ/ቤት ተቀጣሪ 4. ገበሬ 5. የቤት እመቤት 6. ሌላ ካለ ይገለፅ -------- |  |

**ክፍል ሶስት የቅድመ ወሊድ ድባቴ (Depression) መጠይቆች**

| **ተ.ቁ** | **ጥያቄዎች** | **መልስ ሊሆኑ የሚችሉ አማራጮች** | **ምርመራ** |
| --- | --- | --- | --- |
| 3.01 | ባለፈው ሳምነት ዉስጥ የነገሮችን አስቂኝ ሁኔታ በማየት ለመሳቅ ችለዉ ነበር? | 1. በፊት ሳደርግ እንደነበረው 2. እንደበፊቱ አይሆንም 3. በጭራሽ እንደበፊቱ አይሆንም 4. በጭራሽ አልስቅም |  |
| 3.02   | ባለፈዉ ሳምንት ዉስጥ በደስታ/በናፈቆት/ ሲጠባበቁ ነበር? | 1. በፊት የማደርገውን ያህል 2. በፊት ከማደርገው አነስ ያለ 3. በእርግጥ በፊት ከማደርገው ያነስ 4. በጭራሽ አልችልም |  |
| 3.03 | ባለፈዉ ሳምንት ዉስጥ አንደንድ ሁኔታዎች ሳይሳኩ ሲቀሩ ያለአግባብ ራስዎን ይወቅሱ ነበር? | 3-አዎን አብዛኛዉን ጊዜ  2-አዎን አንደንድ ጊዜ  1-አዎን በጣም ጥቅት ጊዜ  0-በፍጹም አልነበረም |  |
| 304 | ባለፈዉ ሳምንት ዉስጥ ያለ በቂ ምክንያት ሲጨነቁ ወይም ሲሰጉ ነበር? | 1. በጭራሽ አልተጨነቅኩም 2. ከቁጥር ለማይገባ ጊዜ 3. አዎን፤ አልፎ አልፎ 4. አዎን፤ አብዛኛዉን ጊዜ |  |
| 3.05 | ባለፈዉ ሳምንት ዉስጥ ያለ በቂ ምክንያት የመፍራት፤የድንጋጤ፤ የመሸበር ስሜት ይሰማዎት ነበር? | 3-አዎን አብዛኛዉን ጊዜ  2-አዎን አልፎ አልፎ  1-አዎን በጣም ጥቅት ጊዜ  0-በፍጹም አይሰማኝም |  |
| 3.06 | ባለፈዉ ሳምንት ዉስጥ ነገሮት ሁሉ ከአቅምዎ በላይ ይሆንብዎት ነበር? | 3-አዎን፤ አብዛኛውን ጊዜ ሁኔታዎችን መቋቋም አልችልም 2-አዎን፤ አልፎ አልፎ ሁኔታዎችን መቋቋም አልችልም 1-አዎን፤ አብዛኛውን ጊዜ ሁኔታዎችን መቋቋም ችያለሁ 0-አዎን፤ በፊት እንደማደርገው ሁኔታዎችን መቋቋም ችያለሁ |  |
| 3.07 | ባለፈዉ ሳምንት ዉስጥ ደስታ ከማጣት የተነሳ የእንቅልፍ ችግር ነበረብዎት? | 3-አዎን አብዛኛዉን ጊዜ  2-አዎን አልፎ አልፎ  1-የለም በጣም ጥቅት ጊዜ  0-በጭራሽ አልተቸገርኩም |  |
| 3.08 | ባለፈዉ ሳምንት ዉስጥ የብስጭት ወይም የመሪር ሃዘን ስሜት ተሰምትዎት ነበር? | 3-አዎን አብዛኛዉን ጊዜ  2-አዎን አልፎ አልፎ  1-የለም በጣም ጥቅት ጊዜ  0-በጭራሽ አልተሰማም |  |
| 3.09 | ባለፈዉ ሳምንት ዉስጥ ደስታ ከማጣት የተነሳ አልቅሰዉ ነበር? | 3-አዎን፤ አብዛኛውን ጊዜ  2-አዎን፤ በመጠኑ ብዉን ጊዜ  1-አዎን አልፎ አልፍ ብቻ  0-በጭራሽ አላለቀስኩም |  |
| 3.10 | ባለፈዉ ሳምንት ዉስጥ እራስዎ ላይ ጉዳት ለማድረስ አስበዉ ነበር? | 3-አዎን አብዛኛዉን ጊዜ  2-አዎን አልፎ አልፎ  1-አምብዛም አስቤ አላውቅም  0-በጭራሽ አላሰብኩም |  |

**ክፍል አራት የማህበረተሰብ ድጋፍ የሚያመላክቱ መጠይቆች**

| 4.01 | **ከፍተኛ ችግር በሚያገጥምብዎት ወቅት ምን ያህል ሰዎች በጣም ይቀርብዎታል?** | የተሰጠው ነጥብ |  |
| --- | --- | --- | --- |
|  | ምንም ሰው አይቀርበኝም | 1 |  |
|  | አንድ ወይም ሁለት | 2 |  |
|  | ከሶስት እስከ አምስት | 3 |  |
|  | ስድስትና ከዚያ በላይ | 4 |  |
| 4.02 | **እርስዎ በሚየደርጉት ነገር ላይ ሰዎች ምን ያህል ትኩረት ይሰጣሉ?** |  |  |
|  | ከፍተኛ ትኩረትና ፍላጎት ይሰጣሉ | 5 |  |
|  | የተወሰና ትኩረትና ፍላጎት ይሰጣሉ | 4 |  |
|  | እርግጠኛ አይደለሁም | 3 |  |
|  | ትንሽ ትኩረትና ፍላጎት ይሰጣሉ | 2 |  |
|  | ምንም ትኩረትና ፍላጎት አይሰጡም | 1 |  |
| 4.03 | **ከጎሮቤት ተግበራዊ እርደታ በሚፈልጉበት ወቅት እንዴት ያገኛሉ?** |  |  |
|  | በጣም በቀላሉ | 5 |  |
|  | በቀላሉ | 4 |  |
|  | ይቻላል | 3 |  |
|  | ይከብዳል | 2 |  |
|  | በጣም ይከብዳል | 1 |  |

**ክፍል አምስት ፡- የስነ- ተዋልዶና አንደንድ የጤና ችግሮችን ታሪክ የሚያመላክቱ መጠይቆች**

| **ተ.ቁ** | **ጥያቄዎች** | **የተሰጡ ምላሾች /መልሶች** | **ምርመራ** |
| --- | --- | --- | --- |
| 5.01 | በህይወት የተወለዱ ልጆች በቁጥር ስንት ነው? | **-----------** |  |
| 5.02 | ካረገዝዎት ምን የህል ጊዜ ሆኖል? | 1.----------  2.አላስታዉስም (እርግጠኛ አይደለሁም) |  |
| 5.03 | የመጨረሻ የወር አበባ የመጠዉ/ የያዎት መቼ ነበር? | --------------- | ለማስተወስ የሚረዱ መረጀዎችን ይጠቀሙ ለምሳል የአልተራሳውንድ ዉጤት |
| 5.04 | የሚወልድብዎት ቀን መቼ ነው? | ------------- |  |
| 5.05 | በአሁኑ ጊዜ ስንት ልጆች አሉዎት? | 1. ምንም የለኝም 2. 1-2 3. 3-4 4. 5 እና ከዚያ በላይ |  |
| 5.06 | ይህንን እርግዝና አስብዎት/ አቅድዎት ነበር የረገዙት? | 1. አዎ 2. አይደለም |  |
| 5.07 | ከአሁን ቀደም መዉለጀ ጊዜ ሳይደርስ ወልድዎት ነበር? | 1. አዎ 2. አይደለም |  |
| 5.08 | ከአሁን ቀደም ውርጃ አገጥምዎት ነበር? | 1. አዎ 2. አይደለም |  |
| 5.09 | የታወቀ የስኳር ህመም አለብዎት? | 1. አዎ 2. አይደለም |  |
| 5.10 | የታወቀ የደም ግፊት ችግር አለብዎት? | 1. አዎ 2. አይደለም |  |
| 5.11 | ቅድመ-ወልድ ክትትል አድርግዎት ነበር? | 1. አዎ 2. አይደለም |  |
| 611 | ወደፊት ልጅ ለመዉለድ ይፈልግዎታል? | 1. አዎ 2. አይደለም |  |

**ክፍል ስድስት ፡- ስለአልኮል/ አስካሪ መጠጥ መረጀ የሚሰጡ ጥያቄዎች**

| **ተ.ቁ** | **ጥያቄዎች** | **የተሰጡ ምላሾች /መልሶች** | | | | | | | **ምርመራ** | | | |
| --- | --- | --- | --- | --- | --- | --- | --- | --- | --- | --- | --- | --- |
| 6.01 | በእርግዝናዎ ወቅት ቡና ይጠጣሉ? | 1. አዎን   1. አይደለም | | | | | | |  | | | |
| 6.02 | መልሶ ለ6.01 አዎን ከሆና በቀን ስንት ሲኒ ቡና ይጠጣሉ? | 1. ከ1-3 2. ከ4-7 3. 8 እና ከዚያ በላይ | | | | | | |  | | | |
| 6.03 | በእርግዝናዎ ወቅት ስጋራ ያጬሳሉ? | - 1. አዎን  1. አይደለም | | | | | | |  | | | |
| 6.04 | በእርግዝናዎ ወቅት ስጋራ ጫት ይቅማሉ? | 1. አዎን 2. አይደለም | | | | | | |  | | | |
| 6.05 | በእርግዝናዎ ወቅት ለመጨረሻ አልኮል የጠጥዎት መቼ ነበር?  ***Seታ¨h: ¾SKŸ=Á ካ`Ê‡” ›d¿*** | 1. ከእርግዝና በፊት 2. በእርግዝና ጊዜ 3. ጠጥቼ አለውቅም | | | | | | | አልኮልነት/ አልኮል ማለት፡-   1. 1 ጠርሙዝ ቢራ 2. 1 ብርጭቆ ወይን (140ሚ.ሊ) 3. 1 መለኪያ (40) አረቂ ፣ጂን፣ ውስኪ 4. 1 ብርሌ (200ሚ.ሊ) ጠጅ 5. 1 ጣሳ (330-500ሚ.ሊ) ጠላ፣ኮርፌ | | | |
| 6.06 | በእርግዝናዎ ወቅት አልኮልነት ያለዉ መጠጥ በያምን ያህል ጊዜ ዉስጥ ይጠጥዎታል? | 1. ጠጥቼ አለውቅም 2. በወር አንድ ጊዜ ወይም ያነሰ 3. በወር ከ2-4 ጊዜ 4. በሳምንት 2-3 ጊዜ 5. በሳምንት 4 ጊዜና በላይ | | | | | | |  | | | |
| 6.07 | በእርግዝናዎ ወቅት አልኮልነት ያለዉ መጠጥ በምጠጥብዎት ጊዜ በአንድ ጊዜ ምን ያህል ልጠጡ ይችላሉ? | 1. 1 ወይም 2 2. 3 ወይም 4 3. 5 ወይም 6 4. 7፣8 ወይም 9 5. 10 እና ከዚያ በላይ | | | | | | |  | | | |
| 6.08 | በእርግዝናዎ ወቅት አልኮልነት ያለዉ መጠጥ በቀን ሰድስት/በአንድ ጊዜ ወይም ከዚያ በላይ በያምን ያህል ጊዜ ዉስጥ ልጠጡ ይችላሉ? | 1. ጠጥቼ አላውቅም 2. አልፎ አልፎ (ወር ዉስጥ ከአንድ ጊዜ ያነሳ) 3. በወር አንድ ጊዜ 4. በሳምንት አንድ ጊዜ 5. በየቀኑ ወይም አልፎ አልፎ በማሳለፍ | | | | | | |  | | | |
| 6.09 | በእርግዝናዎ ወቅት አልኮልነት ያለዉ መጠጥ በቀን/በአንድ ጊዜ አራት ወይም ከዚያ በላይ ምን ያህል ጊዜና መቼ ጠጥተዋሉ? | 1. ጠጥቼ አላውቅም 2. በመጀመሪያዎቹ ሶስት ወራት ዉስጥ-------- 3. ከ3-6 ወር በላው ጊዜ ዉስጥ ----------- 4. ከ6-9 ወር በላው ጊዜ ዉስጥ ---------- | | | | | | |  | | | |
| 6.10 | በእርግዝናዎ ወቅት የጠጥዎት የመጠጥ ዓይነትና መጠን | ጠላ | አረቂ | ጠጅ | ኮሮፌ | ቢራ/  ጃምቦ ድራፍት | ወይን | ዉስኪ፣ጂን፣ቮድካ…. | | ብዛቱን ከነ ዕቃዉ ይግለጹ | |  |
|  | 1. በመጀመሪያዎቹ ሶስት ወራት ዉስጥ |  |  |  |  |  |  |  | |  | |  |
|  | 1. ከ3-6 ወር በላው ጊዜ ዉስጥ |  |  |  |  |  |  |  | |  | |  |
|  | 1. ከ6-9 ወር በላው ጊዜ ዉስጥ |  |  |  |  |  |  |  | |  | |  |
| **ክፍል ሰባት፡- በእርግዝና ወቅት አልኮል ለመጣጠት ---ግለሰቡ፣ማህበረሰቡ እና ባህሉ ያላቸዉ አስተዋጽኦ**   \| **ተ.ቁ** \| **ጥያቄዎች** \| **የተሰጡ ምላሾች /መልሶች** \| **ምርመራ** \| \| --- \| --- \| --- \| --- \| \| 7.01 \| ባለቤትዎ አልኮልነት ያላዉ መጠጥ ይጠጣሉ? \| 1. አዎን 2. አይደለም \|  \| \| 7.02 \| ባለቤትዎ አልኮልነት ያላዉ መጠጥ እንድጠጥዎት ያበረታትዎታል? \| 1. አዎን 2. አይደለም \|  \| \| 7.03 \| ጓደኞችዎት አልኮልነት ያላዉ መጠጥ እንድጠጥዎት ያበረታትዎታል? \| 1. አዎን 2. አይደለም \|  \| \| 7.04 \| በእርግዝና ወቅት አልኮል መጣጠት በባህል ተቀባይት አለዉ? \| 1. አዎን 2. አይደለም \|  \| \| 7.05 \| ለእርስዎ አልኮል እንዲትጠጥዎት ከፍተኛ ሚና የሚጨዎታዉ ማን ነዉ? \| 1. እኔ ራሴ 2. ባለቤቴ 3. ጓደኞቼ 4. የለም \|  \| | | | | | | | | | | |  |  |

**ክፍል ስምንት፡- በእርግዝና ወቅት አልኮል መጠጣት ስለሚያስከትለዉ ጉዳት የመረጀ ምንጭ ጥያቄዎች**

| 8.01 | በእርግዝና ወቅት አልኮል መጠጣት ስለሚየመጠዉ ጉዳት ሰምታዋሉ? | 1. አዎን 2. አይደለም | መልሱ አይደለም ከሆና ወደ ተ.ቁ 9.01 ይህዱ |
| --- | --- | --- | --- |
| 8.02 | በእርግዝና ወቅት አልኮል መጠጣት ጉዳት እንደምያስከትል በምን/ያት ሰሙ? | 1. በቴሌቬዥን 2. በሬድዮ 3. ከጤና ባለሙያ 4. ከጓደኛ/ከቤተሰብ | ከአንድ በላይ ምረጫ መምረጥ ይችላል |
| 8.03 | በቅድመ-ወሊድ ክትትል ጊዜ በእርግዝና ወቅት አልኮል መጠጣት ስለሚየመጠዉ ጉዳት ተነግርዎት ነበር? | 1. አዎን 2. አይደለም |  |

**ክፍል ዘጠኝ፡ የከተማ ቤተሰብ አጠቃላይ ገቢ ሁኔታ (Wealth Index)**

| **ተ.ቁ** | **ጥያቄዎች** | **መልስ** | | |
| --- | --- | --- | --- | --- |
| 9.01 | በአሁኑ ሰዓት የሚኖሩበት ቤት ባለቤቱ ማንነው? | 1. እኔ 2. ኪራይ | | 3. የቤተሰብ 4. ሌላካለ...... |
| 9.02 | የሚኖሩበት ቤት ጣሪያ በዋናነት የተሰራው ከምንድንነው? | 1. በቆርቆሮ የተሰራ 2. የሳርቤት | | 1. ከፕላስቲክ የተሰራ 2. ከብረት 3. ሌላ ካለ |
| 9.03 | የሚኖሩበት ቤት ወለል በዋናነት የተሰራው ከምንድንነው? | - - - 1. ከአፈር/አሸዋ       2. ከእንጨት/ከሸንበቆ       3. ከሴራሚክ | | - - - 1. ከሲሚንቶ       2. ከድንጋይ       3. ሌላካለ.............. |
| 9.04 | የሚኖሩበት ቤት የውጨኛው ግድግዳ በዋናነት የተሰራው ከምንድንነው? | 1. ከአፈር/አሸዋ 2. ከሴራሚክ/ ዕጡብ 3. ከሲሚንቶ | | 1. ከድንጋይ 2. ከእንጨት 3. ሌላ ካለ................. |
| 9.05 | በቤታችሁ ውስጥ ምን ያህል ክፍሎች አሉ? | -------------- | | |
| 9.06 | በቤታችሁ ውስጥ ለመኝታ ክፍል የሚሆን ምንያህል ክፍሎች ይጠቀማሉ? | -------------- | | |
| 9.07 | ለቤተሰብ አባላት የሚሆን ዋና የመጠጥ ውኃ ምንጭ ምንድንነው? (ከአንድ በላይ መልስ ይቻላል) | - - - 1. የቧንቧ ውሃ       2. ከጥልቅ ጉድጓድ       3. የዝናብ | - - - 1. የምንጭ       2. የወንዝ ውሃ       3. ሌላ ካለ................ | |
| 9.08 | በቤትዎ ውስጥ ምን ዓይነት መጸዳጃ ቤት/ቶች አሉዎት? | 1. በውሃ የሚሰራ መጸዳጃ 2. የጉድጓድ ደረቅ መጸዳጃ | 1. መጸዳጃ ቤት የለም 2. ሌላ ካለ…............. | |
| 9.09 | የእርስዎ መኖሪያ ቤት የኤሌክትሪክ ኃይል አለው? | 1. አዎን | 1. የለም | |
| 9.10 | የምግብ ማብሰያ ቤት ለብቻው አለዎት ? | 1. አዎን | 1. የለም | |
| 9.11 | በቤተሰብዎ ውስጥ ምግብ ለማብስል የምትጠቀሙበት ምንድንነው ? ከአንድ በላይ መልስ ይቻላል፡፡ | 1. የኤሌክትሪክ ኃይል 2. እንጨት/ ገለባ 3. ከሰል | 1. ባዮጋዝ 2. ነጭጋዝ 3. ሌላ ካለ............ | |
| 9.12 | በእርስዎ ቤተሰብ መኖሪያ ቤት የትኞቹ የቤት ዕቃዎች አሉ? ከአንድ በላይ መልስ ይቻላል | 1. የቤት ስልክ 2. ማቀዝቀዣ/ፍሪጅ 3. ሬዲዮ | 1. ቴሌቪዥን 2. የኤሌክትሪክ ምጣድ 3. ዘመናዊ አልጋ 4. ሌላ ካለ-- | |
| 9.13 | ከቤተሰብዎ መካከል የሚከተሉት ዕቃዎች ያለው የቤተሰብ አባል አለ?  ከአንድ በላይ መልስ ይቻላል | 1. ብስክሌት--- 2. ባጃጅ---- 3. ሞተር ሳይክል--- | 1. መኪና--- 2. በእንስሳት የሚሳብ ጋሪ---- 3. ሌላ ካለ---- | |
| 9.14 | ቤተሰቡ አባላት ውስጥ የሞባይል ስልክ ያለው አለ? | 1. ስማርት---- | 1. ስማርትያልሆነ---- 3. የለም | |
| 9.15 | የቤተሰቡ ዋና የገቢ ምንጭ ምንድንነው? | 1. ግብርና 2. ወርሀዊ ደሞዝ 3. ንግድ | 1. የቤተሰብ እርዳታ 2. የቀን ስራ 3. ሌላ………… | |
| 9.16 | ቤተሰቡ ውስጥ የባንክ/ማይክሮ ፋይናንስ አካውንት ያለው አለ? | 1. አዎን፤ስንት ------- 2. የለም | | |

**ጥያቄዎቹን በመመለስ ስለተባበሩኝ ከልብ አመስግናለሁ !!!**
